# Greenhouse gas emissions and water footprints of typical dietary patterns in India

**DOI:** 10.1016/j.scitotenv.2018.06.258

**Published:** 2018-12-01

**Authors:** Rosemary F. Green, Edward J.M. Joy, Francesca Harris, Sutapa Agrawal, Lukasz Aleksandrowicz, Jon Hillier, Jennie I. Macdiarmid, James Milner, Sylvia H. Vetter, Pete Smith, Andy Haines, Alan D. Dangour

**Affiliations:** a Department of Population Health, London School of Hygiene & Tropical Medicine, Keppel Street, London WC1E 7HT, UK; b Leverhulme Centre for Integrative Research on Agriculture and Health (LCIRAH), 36 Gordon Square, London WC1H 0PD, UK; c Public Health Foundation of India, Delhi NCR, Plot No. 47, Sector 44, Institutional Area, Gurgaon 122002, Haryana, India; d Royal (Dick) School of Veterinary Studies, University of Edinburgh, Easter Bush, Midlothian EH25 9RG, UK; e Rowett Institute of Nutrition and Health, University of Aberdeen, Aberdeen AB25 2ZD, UK; f Department of Social and Environmental Health Research, London School of Hygiene & Tropical Medicine, 15-17 Tavistock Place, London WC1H 9SH, UK; g Institute of Biological and Environmental Sciences, University of Aberdeen, Aberdeen, AB24 3UU, UK

**Keywords:** Sustainability, India, Greenhouse gas emissions, Water footprint, Agriculture, Dietary pattern

## Abstract

Agriculture is a major contributor to India's environmental footprint, particularly through greenhouse gas (GHG) emissions from livestock and fresh water used for irrigation. These impacts are likely to increase in future as agriculture attempts to keep pace with India's growing population and changing dietary preferences. Within India there is considerable dietary variation, and this study therefore aimed to quantify the GHG emissions and water usage associated with distinct dietary patterns.

Five distinct diets were identified from the Indian Migration Study – a large adult population sample in India – using finite mixture modelling. These were defined as: *Rice & low diversity*, *Rice & fruit*, *Wheat & pulses*, *Wheat, rice & oils*, *Rice & meat*. The GHG emissions of each dietary pattern were quantified based on a Life Cycle Assessment (LCA) approach, and water use was quantified using Water Footprint (WF) data. Mixed-effects regression models quantified differences in the environmental impacts of the dietary patterns.

There was substantial variability between diets: the rice-based patterns had higher associated GHG emissions and green WFs, but the wheat-based patterns had higher blue WFs. Regression modelling showed that the *Rice & meat* pattern had the highest environmental impacts overall, with 0.77 (95% CI 0.64–0.89) kg CO_2_e/*capita*/day (31%) higher emissions, 536 (95% CI 449–623) L/*capita*/day (24%) higher green WF and 109 (95% CI 85.9–133) L/*capita*/day (19%) higher blue WF than the reference *Rice & low diversity* pattern.

Diets in India are likely to become more diverse with rising incomes, moving away from patterns such as the *Rice & low diversity* diet. Patterns such as the *Rice & meat* diet may become more common, and the environmental consequences of such changes could be substantial given the size of India's population. As global environmental stress increases, agricultural and nutrition policies must recognise the environmental impacts of potential future dietary changes.

## Highlights

• India's food system produces large environmental impacts but these vary by diets.
• We explored differences in GHG emissions and water use of Indian dietary patterns.
• Rice-based diets had higher emissions but wheat-based diets used more water.
• Diets with more animal products had the highest environmental impacts overall.
• Diversifying diets in India may have severe consequences for environmental impacts.

## Graphical abstract

**Figure:**
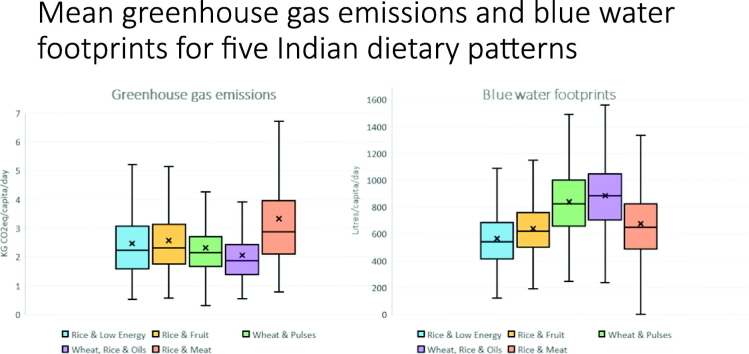

## 1 Introduction

Due to its large population and accelerated pace of development, India is now the world's third largest emitter of anthropogenic greenhouse gases (GHG) (http://cait.wri.org) and the world's largest user of fresh water (Hoekstra and Chapagain, 2007). The food system is a key contributor to both of these environmental impacts: India's agricultural sector is responsible for 22% of the country's total GHG emissions, second in quantity only to the energy sector (71%, http://cait.wri.org), while ~90% of fresh water use is for the purpose of agricultural irrigation (FAO, 2016). The per capita environmental impacts of diets in India are currently lower than those of many high income countries (HICs) due in part to habitually low consumption of animal source foods (Pathak et al., 2010; Scarborough et al., 2014). However, this relatively low per capita impact may change due to the ‘nutrition transition’ away from cereal-dominated diets towards diets high in animal-source and highly-processed foods, at least among some segments of the population (Misra et al., 2011).

Various metrics have been developed to assess the environmental impacts of food systems and diets. These include the quantification of GHG emissions and water usage of food items, food production systems and diets through Life Cycle Assessment (LCA) and Water Footprint (WF) Assessment (Boulay et al., 2013; Pradhan et al., 2013). Emissions of GHGs are typically quantified using a standard metric of mass of CO_2_ equivalent (CO_2_e) associated with the production, processing and retail of food items. An additional factor may be included to account for food waste. In WF assessment, freshwater use is distinguished between rain water that cycles via the topsoil and evapotranspiration (green WF), ground and surface water used for irrigation (blue WF) and a measure of water pollution (grey WF). Irrigation is widespread in India (Government of India, 2016), allowing farmers to grow 2–3 crops per year and increasing food security. However, decades of net extraction of groundwater have led to falling water tables in some parts of India coupled with concerns about groundwater quality, for example due to salination and contamination with arsenic (MacDonald et al., 2016; Rodell et al., 2009). The blue WF of diets may therefore be of particular interest.

To date, the majority of studies on the environmental sustainability of diets have considered diets in high income countries (HICs), with many suggesting that reduced consumption of animal-source and highly processed foods could lead to reductions in the GHG emissions and/or WFs associated with diets (Garnett, 2011; Berners-Lee et al., 2012; Macdiarmid et al., 2012; Green et al., 2014; Scarborough et al., 2014; Tilman and Clark, 2014; Aleksandrowicz et al., 2016). Dietary changes have also been proposed that could deliver both environmental and health benefits (Friel et al., 2009; Aston et al., 2012; Green et al., 2014; Milner et al., 2015; Gephart et al., 2016), although a recent systematic review of the evidence (almost entirely from HICs) has indicated that not all changes will be beneficial and that trade-offs may be required between different environmental impacts such as GHGs and water use (Aleksandrowicz et al., 2016). Additionally, the dietary changes that have been proposed may not be relevant to low or middle income countries (LMICs) due to the different food systems, patterns of consumption and nutritional challenges. Several studies have quantified the WF of agriculture in India (e.g. Hoekstra and Chapagain, 2007; Kampman, 2007; Jayaram and Mathur, 2015) and one study has quantified the GHG emissions of broad food groups commonly occurring in Indian diets (Pathak et al., 2010) suggesting that GHG emissions of diets ranged from ~0.6 kg CO_2_e/*capita*/day for a vegetarian diet to ~1 kg CO_2_e/*capita*/day for a non-vegetarian diet. However, data are lacking on the GHG emissions and WFs associated with existing typical dietary patterns, and no previous research has combined multiple environmental impacts into a single study.

The present study set out to quantify the GHG emissions and WFs associated with typical dietary patterns in India, based on existing dietary data. The analysis was based on a pre-defined set of five distinct dietary patterns that capture some of the regional diversity in Indian diets (Joy et al., 2017), enabling an exploration of the levels of change in environmental impacts that could be expected if consumers of the more traditional patterns were to adopt other diets as part of a nutrition transition.

## 2 Methods

We quantified the GHG emissions and WFs associated with distinct dietary patterns in India by combining various data sources on food consumption, GHG emissions and WFs.

### 2.1 Food consumption data and dietary patterns

Typical dietary patterns were previously defined in a population of 7067 Indian adults recruited into the Indian Migration Study (IMS), conducted during 2005–2007, which provides a unique source of high quality dietary intake data (Joy et al., 2017). Detailed methodology regarding IMS participant recruitment, generation of dietary data and definition of distinct dietary patterns is described elsewhere (Reddy et al., 2006; Joy et al., 2017). Briefly, the IMS recruited factory workers who had migrated to one of four Indian cities (Bangalore, Hyderabad, Lucknow and Nagpur) along with their spouses and rural-dwelling siblings, as well as a 25% sample of urban non-migrants. The majority of IMS respondents were male (57%), married (88%) and follow the Hindu religion (91%). Almost half of the respondents were from the southern region of India, with 30% from the north, 20% from the west, and only 2% from the east. The mean age of the respondents was 41 years (range 17–76 years).

Dietary intake was quantified using an interviewer-administered food frequency questionnaire (FFQ) comprising 184 dishes and food items. These were disaggregated into 199 individual foods and assigned to 36 food groups (Appendix A, Table A1) and dietary patterns were defined through finite mixture modelling (Joy et al., 2017). Five distinct patterns were identified and named after their predominant staple grain plus one other identifying feature: *Rice & low diversity*; *Rice & fruit*; *Wheat & pulses*; *Wheat, rice & oils*; and *Rice & meat* (Appendix A, Table A2). The patterns were regionally distributed, with rice-based patterns consumed largely in the south and wheat-based patterns in the north and west. The patterns captured other socio-demographic characteristics, for example the *Rice & low diversity* pattern was consumed predominantly by adults with little formal education located in rural settings and was thus identified in this analysis as being a reference diet due to its lack of diversity, while the *Rice & fruit* pattern was consumed predominantly by adults with more formal education living in urban areas.

### 2.2 Quantification of environmental impacts

The majority of food consumed in India is domestically produced (Appendix A, Table A3). For example, 216,517 t of cereals were available for consumption in 2013 in India of which only 113 t (0.1%) were imported. Similarly, 133,443 t of milk were available for consumption, of which 18 t (0.01%) were imported (FAO, 2016). There were two food groups for which this was not the case: for both tree nuts and vegetable oils >50% of the available food was imported. However, these food groups comprise a very small amount of the total diet by weight. Emissions of GHGs and WFs were therefore quantified using India-specific production data.

Emissions of GHGs across the life cycle (kg CO_2_e/kg food) for each of the 36 food groups were derived from published data. Where possible, India-specific data were used, but where these were not available for particular food groups, data were extrapolated from other foods and/or countries (Appendix A, Table A4). For each group, we combined estimates of emissions from food production, storage, processing, transport, cooking and packaging (Foster et al., 2006; Audsley et al., 2009; FAO, 2011; Pathak et al., 2010; Williams et al., 2006; Röös et al., 2015; Vetter et al., 2017). An additional factor for emissions due to food waste was added, which was quantified as the product of emissions from all other stages and the proportion of different food groups typically wasted at all stages from production to consumption using FAO estimates for South and South East Asia (FAO, 2016). Finally, FAO Technical Conversion Factors were applied to account for waste of each food item at the production stage, the proportion of the food item that is edible and the value that accrues to the particular food item rather than by-products (e.g. the value fraction for butter accounts for the whey also produced from the milk). Emissions from the food production process for most food groups were quantified using the Cool Farm Tool (https://www.coolfarmtool.org/; Hillier et al., 2011; Vetter et al., 2017).

We considered green (from precipitation) and blue (from irrigation) WFs in the analysis. We did not include grey WF, because this is a measure of the amount of water polluted rather than water demand. As such, grey WF is measuring a different type of environmental impact and our decision to focus on green and blue WFs is consistent with suggestions in recent literature (Chenoweth et al., 2014). The green and blue WFs (L/g food) of crop-based food groups were derived from published data from the Water Footprint Network (www.waterfootprint.org; Mekonnen and Hoekstra, 2011). The WFs of livestock products in each state were calculated based on methods described in Mekonnen and Hoekstra (2012), i.e. including the indirect WF of feed and the direct water consumption from drinking and service water. The WF of feed was calculated for each livestock category under grazing, industrial and mixed production systems (Mekonnen and Hoekstra, 2012). The Water Footprint Network dataset does not report WFs of aquatic products, so the WFs of prawns and fish were quantified based on the WF of fish feed ingredients in each state. Carp account for >90% of freshwater fish production in India so the WF of fish was based on major carp species. The 184 IMS food items were matched to state-level data on the WF of food products, then aggregated to 36 food consumption groups by calculating the mean WF of constituent items, weighted by their relative contribution to total food group consumption across the IMS population. National WFs for each food group were quantified by taking the mean of state-level data weighted by land size (Office of Registrar General of India, Ministry of Home Affairs, 2011) (Appendix A, Table A5). Full details of the estimation process can be found in Harris et al. (2017).

### 2.3 Statistical analysis of relationships between dietary patterns and environmental impacts

Having estimated the GHG emissions and WFs associated with each food group, we used these estimates to quantify the total GHG emissions and WFs associated with the daily diet of each participant in the IMS. This also provided us with an estimate of the mean GHG emissions and WF associated with each of the five dietary patterns.

To explore the relationships between different dietary patterns and environmental impacts of diets before and after adjustment for energy content of the diet, we constructed mixed effects linear regression models with dietary pattern as a categorical independent variable and the three different environmental impacts (GHG emissions, green WF and blue WF) as continuous dependent variables. We included a random effect to account for clustering by family (IMS being originally a study of sibling pairs), and fixed effects to describe the dietary patterns. In the adjusted models we included total dietary energy as an additional (continuous) independent variable.

For all analyses the *Rice & low diversity* pattern was used as the reference diet against which the other four patterns were compared, due to its low energy content and lack of diversity. The regression coefficients for the other four patterns were then used to explore the changes to environmental impacts that would occur if consumers of the reference diet were to adopt one of the other dietary patterns. Statistical analyses were conducted using STATA (Version 13; StataCorp LP, College Station, TX, USA).

## 3 Results

### 3.1 Environmental impacts of Indian foods

**Fig. 1:**
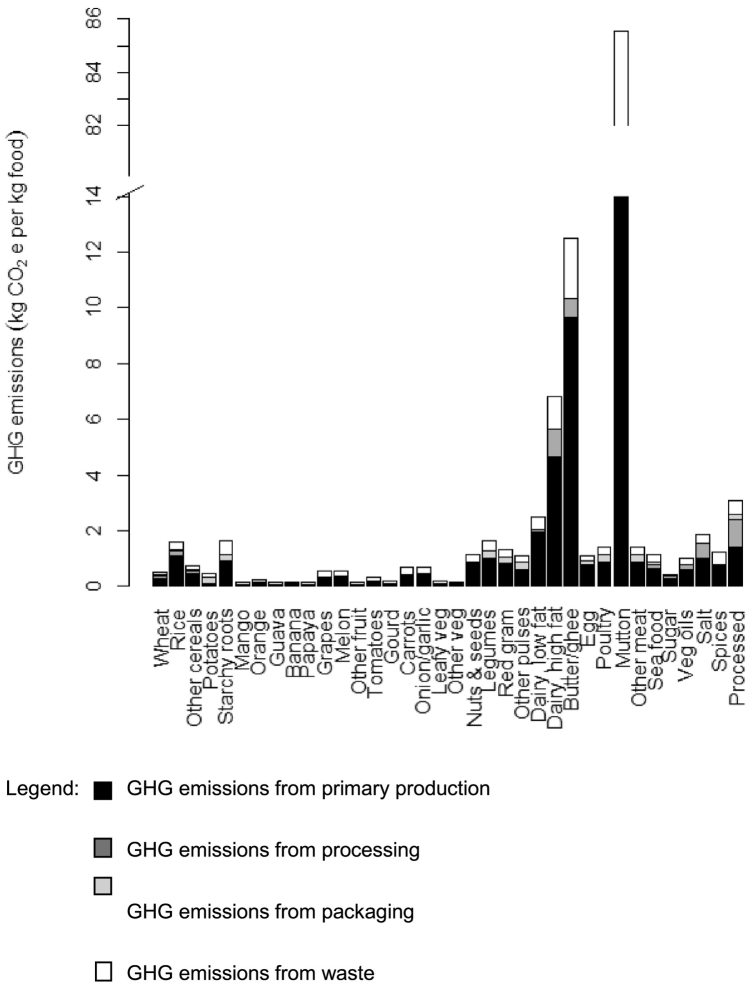
Greenhouse gas (GHG) emissions (kg CO_2_e per kg) from selected production stages for 36 food groups identified in the Indian Migration Study. Legend: ■ GHG emissions from primary production. GHG emissions from processing. GHG emissions from packaging. GHG emissions from waste.

Emissions of GHGs were highly variable across the 36 food groups (Fig. 1). Mutton, butter and high fat dairy products had the greatest emissions *per* kg, followed by the “other” (mostly highly processed) food group. Primary production tended to contribute by far the largest share of total GHG emissions, although in some foods (such as dairy and highly processed foods) processing and packaging also contributed substantially.

Three of the 36 food groups contributed nearly 70% of all GHG emissions from food: low fat dairy (mostly milk), mutton and rice. Milk contributed 35% of GHG emissions, mutton contributed 23%, and rice contributed 11% of GHG emissions. The remaining 33 food groups all contributed <5% of GHG emissions each.

**Fig. 2:**
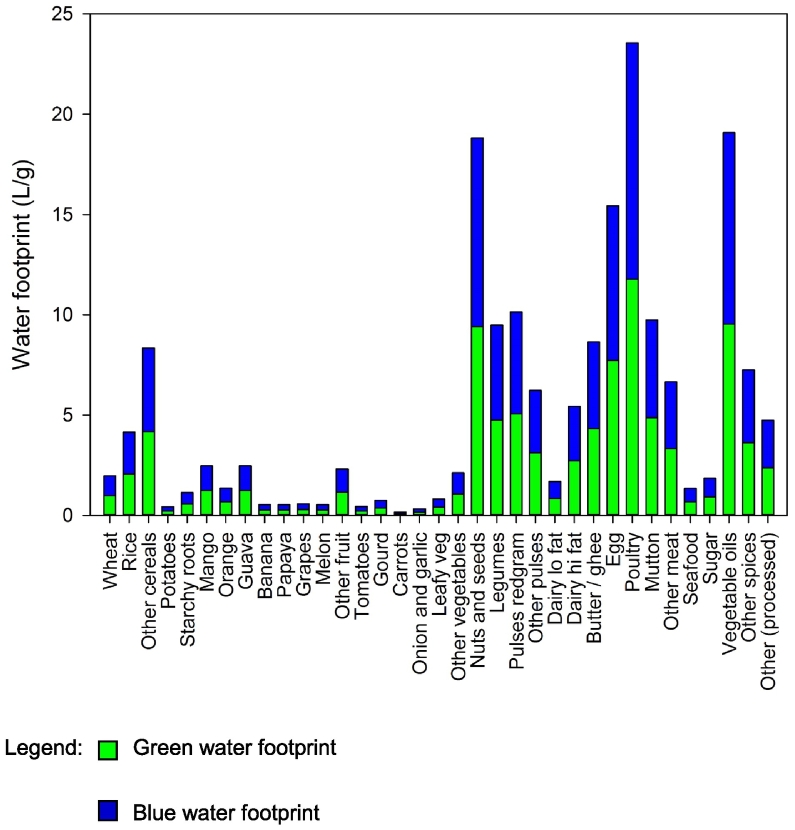
Green and blue water footprints (L/g) of 36 food groups identified in Indian Migration Study Legend:  Green water footprint. Blue water footprint.

WFs of food groups were also highly variable (Fig. 2), with the greatest green and blue WFs per g for animal products, vegetable oils and nuts and seeds. Pulses and legumes had high green WFs while sugar cane and wheat had high blue WFs.

The vegetable oils food group contributed 18% of total green WF across the IMS population, while rice, dairy and wheat comprised 15%, 13%, and 7%, respectively. When meat food groups were combined, they contributed just 6% of green WF. Wheat comprised 31% of total blue WF across the IMS population, rice contributed 19%, and dairy contributed 15%. Meat food groups combined contributed <5% of blue WF.

### 3.2 Indian dietary patterns and their environmental impacts

The five dietary patterns identified using IMS data have been described in previous work (Joy et al., 2017; see also Appendix A, Table A2). Briefly, we found that three diets were rice-based (mainly consumed by individuals in southern India) and the remaining two were wheat-based (mainly consumed by individuals in northern India). The *Rice & low diversity* diet was the least diverse and the lowest in calories, and we therefore used this as a reference diet against which the other patterns were compared.

The mean GHG emissions associated with the Indian diets we examined were 2.4 ± 1.3 kg CO_2_e/*capita*/day (mean ± standard deviation; Table 1). However, this was variable between dietary patterns, ranging from 2.1 ± 1.2 kg CO_2_e/*capita*/day for the *Wheat, rice & oil* pattern to 3.3 ± 1.9 kg CO_2_e/*capita*/day for the *Rice & meat* pattern. Rice-based dietary patterns had ~25% greater emissions than wheat-based patterns.

**Table 1:** Greenhouse gas (GHG) emissions of typical Indian dietary patterns. Values are mean (standard deviation) of individuals by dietary pattern.

Dietary pattern	*N* (%)	Energy consumption	Total GHG emissions
		Mean (SD)	
		kcal/day	kg CO_2_e/*capita*/day
Rice & low diversity	1339 (20)	2369 (760)	2.47 (1.30)
Rice & fruit	1505 (22)	2762 (813)	2.57 (1.21)
Wheat & pulses	1953 (29)	3027 (856)	2.30 (1.04)
Wheat, rice & oils	1462 (22)	3344 (868)	2.05 (1.20)
Rice & meat	516 (8)	2723 (827)	3.31 (1.88)
All	6775 (100)	2883 (892)	2.42 (1.28)

The green and blue WFs of the overall IMS population were 2531 ± 885 and 737 ± 263 L/*capita*/day and were also highly variable between dietary patterns (Table 2). The *Rice & meat* pattern had the greatest green WF with 2776 ± 1032 L/*capita*/day, while the *Wheat, rice and oils* pattern had the greatest blue WF. Proportionately larger differences between dietary patterns were found for blue WF, where the wheat-based diets had larger WFs.

**Table 2:** Green and blue water footprints (WF) of typical Indian dietary patterns.

Dietary pattern	*N* (%)	Energy consumption	Water footprint	
		Mean (SD)		
			Green	Blue
		kcal/day	L/*capita*/day	
Rice & low diversity	1339 (20)	2369 (760)	2209 (797)	566 (208)
Rice & fruit	1505 (22)	2762 (813)	2683 (924)	640 (191)
Wheat & pulses	1953 (29)	3027 (856)	2492 (820)	836 (252)
Wheat, rice & oils	1462 (22)	3344 (868)	2636 (864)	883 (254)
Rice & meat	516 (8)	2723 (827)	2776 (1032)	677 (248)
All	6775 (100)	2883 (892)	2531 (885)	737 (263)

### 3.3 Quantifying differences in environmental impacts between dietary patterns

Compared to the reference dietary pattern (*Rice & low diversity*), consumption of all other dietary patterns except the *Rice & meat* pattern was associated with significantly lower per capita GHG emissions (Table 3). The wheat-based diets tended to have lower GHG emissions than the rice-based patterns. For example, consumers of the *Wheat, rice and oils* pattern had 0.39 kg CO_2_e/*capita*/day (16%) lower emissions than the reference pattern, while consumers of the *Rice and meat* pattern had 0.77 kg CO_2_e/*capita*/day (31%) higher emissions than the reference pattern. The difference between rice and wheat based patterns was accentuated after adjustment for total energy intake, so that consumers of the *Wheat, rice and oils* pattern had 0.44 kg CO_2_e/*capita*/day (18%) lower emissions than the reference pattern after adjustment. The higher emissions of the *Rice and meat* pattern were slightly attenuated after adjustment for total energy intake, but consumers of this pattern still had 0.51 kg CO_2_e/*capita*/day (21%) higher emissions than those of the reference pattern.

**Table 3:** Mixed effects linear regression analysis to quantify differences in environmental impacts of dietary patterns in India.

Dietary pattern	Difference relative to reference diet, mean (95% CI)			
	Unadjusted energy	*p*	Adjusted for total	*p*
Total GHG emissions in kg CO_2_e/*capita*/day				
Rice & low diversity (ref)	–	–	–	–
Rice & fruit	0.08 (−0.01, 0.18)	0.08	−0.21 (−0.29, −0.13)	<0.001
Wheat & pulses	−0.18 (−0.28, −0.08)	<0.001	−0.44 (−0.52, −0.36)	<0.001
Wheat, rice & oils	−0.39 (−0.49, −0.29)	<0.001	−0.95 (−1.04, −0.86)	<0.001
Rice & meat	0.77 (0.64, 0.89)	<0.001	0.51 (0.40, 0.62)	<0.001

Green water footprint in L/*capita*/day				
Rice & low diversity (ref)	–	–	–	–
Rice & fruit	377 (312, 442)	<0.001	66.5 (32.7, 100)	<0.001
Wheat & pulses	233 (166, 301)	<0.001	−44.5 (−78.7, −10.3)	0.011
Wheat, rice & oils	364 (292, 436)	<0.001	−247 (−284, −210)	<0.001
Rice & meat	536 (449, 623)	<0.001	266 (220, 311)	<0.001

Blue water footprint in L/*capita*/day				
Rice & low diversity (ref)	–	–	–	–
Rice & fruit	63.0 (45.6, 80.4)	<0.001	−28.8 (−36.5, −21.0)	<0.001
Wheat & pulses	261 (243, 278)	<0.001	179 (170, 186)	<0.001
Wheat, rice & oils	302 (283, 321)	<0.001	125 (117, 134)	<0.001
Rice & meat	109 (85.9, 133)	<0.001	31.7 (21.4, 42.0)	<0.001

All four other dietary patterns had higher green WFs than the *Rice & low diversity* pattern (Table 3). Compared to the reference pattern, the *Wheat, rice & oils* pattern had a green WF that was 364 L/*capita*/day (17%) higher, while the *Rice & meat* pattern was 536 L/*capita*/day (24%) higher. After adjustment for total energy, however, the wheat-based patterns were associated with lower green WFs, so that the *Wheat, rice & oils* pattern had a green WF 247 L/*capita*/day (11%) lower than the reference pattern. The largest green WF was still associated with the *Rice & meat* pattern, which was 266 L/*capita*/day (12%) higher than the reference dietary pattern.

A similar result was seen for blue WF before adjusting for total energy intake, with all other patterns demonstrating a higher blue WF than the *Rice & low diversity* pattern (Table 3). However, differences were much larger for the wheat-based patterns, such that the *Wheat, rice & oils* pattern had a blue WF 302 L/*capita*/day (53%) higher than the reference pattern, whereas the *Rice and fruit* pattern was only 63 L/*capita*/day (11%) higher. After adjustment for total energy these relationships were again attenuated, and the *Rice & fruit* pattern actually had a lower blue WF per kcal than the reference pattern. The other three dietary patterns, however, still had significantly higher blue WFs than the *Rice & low diversity* pattern.

## 4 Discussion

### 4.1 Principal findings

The distinct dietary patterns explored in this study have markedly different environmental implications. In regression analysis, the rice-based patterns had greater GHG emissions than the wheat-based patterns, principally due to CH_4_ emissions from flooded rice crop production. Emissions associated with the production of animal source foods were generally greater per kg food produced than those of plant-based foods, and the dietary pattern with the greatest emissions was the *Rice & meat* pattern. The rice-based patterns also had higher green WFs, since rice is typically grown in the *Kharif* (monsoon) season and the majority of its water requirements are met by rainfall. Conversely, the wheat-based patterns had the greatest blue WFs, since wheat is typically grown in the *Rabi* (dry) season, relying on residual soil moisture and irrigation.

As India continues in its nutrition transition, people currently consuming the *Rice & low diversity* pattern (which had low energy and diversity, and was consumed most commonly by rural dwellers and those who owned agricultural land (Joy et al., 2017)) will be likely to adopt more diverse diets that may resemble the other dietary patterns we identified. Our regression analyses indicate that adoption of one of the wheat-based patterns or the *Rice & fruit* pattern instead of the reference pattern would result in reduced per capita GHG emissions but an increased green and blue WF. However, if these patterns were adopted without the increase in overall energy content implied by current consumption patterns, the wheat-based patterns would be lower in green WF than the reference diet, while the *Rice & fruit* diet would be lower in blue WF. This indicates the importance of overall dietary energy intake as well as dietary pattern for the environmental consequences of diets. Adoption of the *Rice & meat* pattern would result in an increase in all three measures of environmental impact even if dietary energy intake was not increased.

It is therefore clear that while some dietary patterns (such as the *Rice & meat* pattern) are overall less desirable from an environmental perspective, others require consideration of the trade-offs between GHG emissions and water use, and also between use of irrigation (blue WF) and rainwater (green WF). This is in addition to potential trade-offs with the nutritional content and associated health impacts of diets (Joy et al., 2017). For example, consumers of the *Rice & low diversity* pattern were found to have inadequate fruit and vegetable intake and so some increase in consumption would be desirable for this group, but once total energy intake was adjusted for the *Rice & low diversity* pattern actually had the second highest GHG emissions due to its high rice content. An increase in fruit and vegetable consumption among this group without an associated decrease in rice consumption could therefore lead to a large increase in environmental impacts.

### 4.2 Study strengths and limitations

This is the first study of its kind to assess the environmental impacts of diets in a developing country context, using empirically defined, sub-national dietary patterns. The study was able to evaluate multiple environmental impacts associated with diets in contrast to most pre-existing studies which use GHG emissions as the only measure of environmental impact. There are a number of limitations to the study which must be considered in interpreting the results.

The IMS population used to quantify dietary patterns is not representative of the entire population of India, e.g. the study under-represents the poorest individuals and those living in the East and Northeast of the country, and does not include children. Further study is required to define typical dietary patterns in a nationally representative population. In addition, dietary patterns are rapidly changing among some populations in India, including greater consumption of highly processed foods, and more recent dietary data would enable further investigation of India's continuing transition towards more complex and highly processed diets. Other dietary data sources for India are available (Aleksandrowicz et al., 2017), but these data also have their own limitations, for example by quantifying consumption at household level rather than individual level. It was also assumed that foods consumed by IMS participants were produced in India as during the time period of IMS study, the majority of food consumed in India was domestically produced. In future work, it may be necessary to incorporate trade data due to the increasing integration of India's food system with global markets.

There are inherent limitations in the accuracy of GHG and WF estimates of crop and livestock production due to the availability of data at appropriate resolution for multiple input parameters (Vetter et al., 2017; Zhuo et al., 2014). For example, there were no relevant GHG data for 14 of 36 food groups representing 22% of calories. Where possible, India-specific data from other sources were used, or values from similar food groups were used based on author judgement. India-specific data on environmental implications of production were also used where possible. However, there is significant variation in environmental and crop/livestock production factors within India. Sensitivity to production location was tested by matching food consumption to state-level GHG and WF data (Appendix A, Table A5 and Section A1). Re-running the mixed effects regression analyses using state-level rather than national average data resulted in generally larger differences between the reference diet and the other dietary patterns, but substantively the main relationships found were unchanged. However, there is likely to be significant within-state variation in the GHG emissions and WFs associated with food items and these were not investigated due to data limitations. In addition, there are likely to be significant volumes of inter-state trade of food items, and it is not certain whether linking food consumption to state-level production data based on residency location would provide a more accurate match. A mapping of the food system could increase the accuracy with which production and consumption data are matched.

Finally, there are conceptual limitations to the metrics used in this study. For example, the WF metric does not relate to the availability of water in the region of study and therefore does not provide a direct measure of the local impact of water use (Chenoweth et al., 2014). In addition, the green WF may have little relevance for informing water management strategies because it is not accessible for other uses (Ridoutt and Pfister, 2010), although green and blue water use are intrinsically linked, and improving green water productivity is an additional solution to reducing water scarcity (Hoekstra, 2016). However, the WF remains a useful consumption-based indicator of water use (Velázquez et al., 2011) and the green WF remains important for sustainability assessments, including sensitivity to variation in rainfall (Rockström et al., 2009). It is therefore considered that the best possible estimates of both food consumption and environmental impacts were employed using the data currently available. Future research efforts may include additional measures of environmental impact such as land use change, other pollutants e.g. black carbon, biodiversity loss etc., and may also wish to produce combined metrics of multiple environmental impacts, but these were considered beyond the scope of this study.

### 4.3 Comparison with other studies

Pathak et al. (2010) quantified the GHG emissions associated with the production of hypothetical balanced vegetarian and non-vegetarian diets in India, reporting much lower emissions compared to the present study, i.e. ~0.7 kg CO_2_e/*capita*/day for a vegetarian diet, and 1.0 kg CO_2_e/*capita*/day for a non-vegetarian diet. The present study tended to produce systematically larger estimates of GHG emissions from crops and livestock products, because an attempt was made in this study to account for emissions from the full life-cycle of the product and also to account for waste at the production and household levels, as well as the inedible portions of food products (Appendix A, Table A4). In contrast, Pathak et al. accounted for primary production, processing, transport and preparation only, and it is therefore expected that their GHG emissions estimates would be lower.

To date, most studies of the sustainability of diets have considered diets in HICs and focused on GHG emissions as the sole measure of environmental impact. The per capita GHG emissions of diets in India are generally lower than those reported in HICs. For example, GHG emissions of 5.9 ± 2.0 and 5.7 ± 1.8 kg CO_2_e/*capita*/day were reported for men and women consuming typical meat-containing diets in the UK, i.e. ~3 g CO_2_e/kcal (Scarborough et al., 2014). This compares to 3.3 ± 1.9 kg CO_2_e/capita/day in the *Rice & meat* pattern. Furthermore, many individuals in India prefer vegetarian diets with reported meat and fish consumption among the IMS population of 27 ± 42 g/*capita*/day (Joy et al., 2017). Thus, GHG emissions associated with diets in the overall IMS population was 2.4 ± 1.3 kg CO_2_e/*capita*/day which is more similar to vegetarian diets in the UK, i.e. 3.8 kg CO_2_e/*capita*/day (Scarborough et al., 2014). However, it is difficult to make direct comparisons between studies as they are likely to use different system boundaries.

The green and blue WFs associated with diets among the IMS population were 2531 L/*capita*/day and 737 L/*capita*/day respectively, which compares to 3206 L/*capita*/day and 215 L/*capita*/day for existing diets in Western Europe (Vanham et al., 2013). However, the blue WF contributed 23% to the combined blue and green WF of the diets of the IMS population compared to 7% in Western European vegetarian diets. This reflects the greater reliance on irrigation of food production in India compared to Europe.

### 4.4 Policy relevance and research needs

The variation in GHG emissions and WFs between dietary patterns may inform efforts to quantify future environmental implications of dietary choices and opportunities to minimise environmental impacts, either through influencing dietary choices or through adapting production. Dietary preferences in India are changing due to factors including increasing disposable incomes, urbanisation and globalisation. Many countries have experienced a ‘nutrition transition’ typified by increasing consumption of animal products, edible oils and sugar-sweetened beverages and decreasing consumption of cereals and pulses (Drewnowski and Popkin, 1997; Popkin et al., 2012). However, vegetarianism is common in India for religious and cultural reasons, and while rising meat consumption was a major feature of China's nutrition transition (Kearney, 2010), India's transition may instead be typified by rising consumption of milk and dairy products. For example, energy from milk consumption increased by ~30% per capita in India between 1990 and 2013 (FAO, 2016).

The *Rice & low diversity* pattern used as the reference pattern in our analyses was consumed by 20% of the study population. Its consumers had on average the least formal education and 57% resided in rural areas compared to 37% of the IMS population as a whole, being also the most likely to own agricultural land (Joy et al., 2017). This pattern therefore appears closest to a traditional Indian diet (at least in rice-dominated areas), and it is likely that in future consumers of this diet may adopt diets closer to the other four patterns identified.

As the dietary patterns showed a strong link with region of residence in their preference for rice or wheat as a staple grain, the diets adopted in the shift away from more traditional and low-diversity consumption patterns are likely to be strongly dependent on region. However, the evidence from this study shows that the pattern of choice is likely to have significant implications for the environmental impacts of the food system. More widespread adoption of wheat-based diets would be likely to reduce GHG emissions and green WFs, but increase blue WFs. If irrigation strategies could be better planned and delivered, this would lead to a notable overall environmental benefit over the adoption of the rice-based diet consumed by wealthier and more highly educated adults (the *Rice & fruit* pattern). It is also notable that diversifying diets away from polished rice may deliver environmental and health co-benefits, including reducing the risk of diabetes (Hu et al., 2012), improving micronutrient intakes and reducing GHG emissions from methane (Rao et al., 2018), but whether such changes to staple foods in the diet would be acceptable to the population is a key question for future research. Diets cannot be considered truly sustainable unless they are culturally so. Future studies may also add other dimensions of sustainability, including resilience to price or weather shocks, affordability and implications for agricultural land area, in order to inform dietary recommendations that support future food security and health.

## 5 Conclusion

This study has shown that the per capita environmental impacts of typical diets in India are relatively low compared to diets in HICs, but also that there is wide variation between different dietary patterns. As diets in India continue to change there is the potential for the environmental impacts of food systems to increase, and the implications for water resources may be particularly vital for future food security and health.

## Funding

This work was supported by the Wellcome Trust ‘Our Planet, Our Health’ programme (Grant number 103932). The Wellcome Trust had no role in the design, analysis or writing of this article.

The Indian Migration Study (IMS) was funded by Wellcome Trust (Grant number GR070797MF).

LA's PhD studentship is funded by the Leverhulme Centre for Integrative Research on Agriculture and Health (LCIRAH). SA is supported by a Wellcome Trust Capacity Strengthening Strategic Award-Extension phase to the Public Health Foundation of India and a consortium of UK universities (WT084754/Z/08/A).

## Appendix A Supplementary data

Supplementary material Image 6
