# Supplementary material for: Greenhouse gas emissions and water footprints of typical dietary patterns in India
Source: Sci Total Environ. 2018 Dec 1;643:1411–8. doi: 10.1016/j.scitotenv.2018.06.258 (PMC6137647; doi:10.1016/j.scitotenv.2018.06.258)
Supplement: Supplementary file 1 — Supplementary material [file mmc1.docx]

**Appendix A – Supplementary Tables**

**Table A1. Composition of food groups.**

| \| **IMS Food Name** \| **Consumption Weighting** \| **Food Group for Analysis** \| \| --- \| --- \| --- \| \| Wheat flour \| 0.90 \| Cereals - wheat \| \| Rava \| 0.06 \|  \| \| Noodles \| 0.00 \|  \| \| Bread \| 0.03 \|  \| \| Dalia \| 0.01 \|  \| \| Rice (polished ) \| 0.93 \| Cereals - rice \| \| Rice flakes \| 0.05 \|  \| \| Puffed rice \| 0.02 \|  \| \| Rice flour \| 0.00 \|  \| \| Ragi flour \| 0.36 \| Cereals - other \| \| Ragi \| 0.23 \|  \| \| Jowar flour \| 0.37 \|  \| \| Bajre ki atta \| 0.02 \|  \| \| Bajra \| 0.01 \|  \| \| Corn flour \| 0.01 \|  \| \| Cornflakes \| 0.00 \|  \| \| Bhagar (panivaragu) \| 0.00 \|  \| \| Milk* \| 0.94 \| Dairy - lo-fat \| \| Curds \| 0.06 \|  \| \| Hung curd \| 0.00 \|  \| \| Khoa* \| 0.44 \| Dairy - hi-fat \| \| Paneer* \| 0.19 \|  \| \| Cream  Ice cream \| 0.00  0.37 \|  \| \| Butter \| 0.16 \| Dairy - butter/ghee \| \| Ghee \| 0.85 \|  \| \| Fish \| 0.85 \| Fish, seafood \| \| Prawn \| 0.15 \|  \| \| Raw mango \| 1.00 \| Fruit - mango \| \| Musambi \| 1.00 \| Fruit - orange \| \| Guava \| 1.00 \| Fruit - guava \| \| Banana \| 1.00 \| Fruit - banana \| \| Papaya \| 1.00 \| Fruit - papaya \| \| Grapes \| 1.00 \| Fruit - grapes \| \| Musk melon \| 0.58 \| Fruit - melon \| \| Watermelon \| 0.42 \|  \| \| Custard apple \| 0.09 \| Fruit - other \| \| Apple \| 0.22 \|  \| \| Zizyphus \| 0.07 \|  \| \| Coconut \| 0.07 \|  \| \| Sapota \| 0.07 \|  \| \| Jamoon \| 0.06 \|  \| \| Pomegranate \| 0.04 \|  \| \| Lime juice \| 0.02 \|  \| \| Sweet lime \| 0.06 \|  \| \| Jack fruit \| 0.07 \|  \| \| Lemon \| 0.03 \|  \| \| Lemon juice \| 0.01 \|  \| \| Pineapple \| 0.02 \|  \| \| Raisins \| 0.04 \|  \| \| Tamarind \| 0.04 \|  \| \| Pears \| 0.01 \|  \| \| Litchis \| 0.01 \|  \| \| Palmyra \| 0.01 \|  \| \| Plums \| 0.01 \|  \| \| Kiwi \| 0.00 \|  \| \| Raw plantain \| 0.01 \|  \| \| Amla \| 0.01 \|  \| \| Dry mango slice \| 0.01 \|  \| \| Peaches  Coconut milk \| 0.01  0.01 \|  \| \| Chicken \| 1.00 \| Meat - poultry \| \| Other poultry \| 0.00 \|  \| \| Egg \| 1.00 \| Egg \| \| Mutton \| 1.00 \| Meat - mutton \| \| Pigeon \| 0.53 \| Meat - other \| \| Liver \| 0.43 \|  \| \| Rabbit \| 0.02 \|  \| \| Salami \| 0.01 \|  \| \| Brain \| 0.01 \|  \| \| Kidney beans \| 0.17 \| Legumes \| \| Cluster beans \| 0.01 \|  \| \| Groundnut \| 0.82 \|  \| \| Cashewnuts \| 0.83 \| Nuts and seeds \| \| Sesame seeds \| 0.09 \|  \| \| Chironji \| 0.05 \|  \| \| Almonds \| 0.03 \|  \| \| Pistachio nut \| 0.00 \|  \| \| Sugar \| 0.83 \| Other - sugar \| \| Sugarcane  Honey  Jaggery \| 0.12  0.00  0.05 \|  \| \| Coca-cola \| 0.35 \| Other \| \| Chocolate \| 0.04 \|  \| \| Local arrack/toddy \| 0.21 \|  \| \| Beer \| 0.12 \|  \| \| Tea powder \| 0.10 \|  \| \| Spirits (whiskey, gin, rum) \| 0.08 \|  \| \| Wine \| 0.02 \|  \| \| Ketchup, tomato sauce \| 0.02 \|  \| \| Horlicks \| 0.01 \|  \| \| Coffee powder \| 0.01 \|  \| \| Pav \| 0.01 \|  \| \| Pizza \| 0.01 \|  \| \| Jam \| 0.01 \|  \| \| Baking powder \| 0.01 \|  \| \| Sago \| 0.00 \|  \| \| Custard powder\corn flour \| 0.00 \|  \| \| Mango pickle masala \| 0.00 \|  \| \| Soya sauce \| 0.00 \|  \| \| Soda \| 0.00 \|  \| \| Lemon Pickle masala \| 0.00 \|  \| \| Kala namak \| 0.00 \|  \| \| Papad khar \| 0.00 \|  \| \| Vinegar \| 0.00 \|  \| \| Red gram dhal \| 1.00 \| Pulses - red gram \| \| Salt \| 1.00 \| Salt \| \| Black gram dhal \| 0.15 \| Pulses - other \| \| Chick peas \| 0.02 \|  \| \| Besan \| 0.12 \|  \| \| Green gram dal \| 0.11 \|  \| \| Bengal gram dhal \| 0.09 \|  \| \| Semi falli (broad bean) \| 0.04 \|  \| \| Kidney Beans \| 0.00 \|  \| \| Masoor dhal \| 0.02 \|  \| \| Black gram \| 0.00 \|  \| \| Black gram dhal flour \| 0.00 \|  \| \| Green gram \| 0.00 \|  \| \| Peas \| 0.45 \|  \| \| Onion \| 0.94 \| Veg – onion and garlic \| \| Spring onions \| 0.00 \|  \| \| Green onion  Garlic \| 0.00  0.06 \|  \| \| Cabbage \| 0.26 \| Leafy veg \| \| Green vegetable (dhantu) \| 0.00 \|  \| \| Gogu  Amaranth \| 0.04  0.12 \|  \| \| Spinach \| 0.57 \|  \| \| Coriander leaves \| 0.01 \|  \| \| Mint leaves \| 0.00 \|  \| \| Jeera \| 0.05 \| Spices - other \| \| Methi seeds \| 0.31 \|  \| \| Turmeric pder \| 0.11 \|  \| \| Red chilly powder \| 0.11 \|  \| \| Mustard \| 0.06 \|  \| \| Sambar powder \| 0.03 \|  \| \| Danya pder \| 0.09 \|  \| \| Kasuri methi \| 0.02 \|  \| \| Red chilly \| 0.02 \|  \| \| Pepper corn \| 0.01 \|  \| \| Coriander seeds \| 0.03 \|  \| \| Dry red chilly \| 0.01 \|  \| \| Poppy seeds \| 0.01 \|  \| \| Cardamom \| 0.01 \|  \| \| Asofoetida \| 0.01 \|  \| \| Cinnamon \| 0.01 \|  \| \| Amchur powder \| 0.00 \|  \| \| Cloves \| 0.00 \|  \| \| Omum \| 0.00 \|  \| \| Bay leaves \| 0.00 \|  \| \| Black cardamom \| 0.00 \|  \| \| Cardamom powder \| 0.00 \|  \| \| Shajeera \| 0.00 \|  \| \| Cumin seed powder \| 0.00 \|  \| \| Mustard powder \| 0.00 \|  \| \| Biriyani powder \| 0.00 \|  \| \| Saunf \| 0.00 \|  \| \| Jaiphal \| 0.00 \|  \| \| Ajwain powder \| 0.00 \|  \| \| Javithri \| 0.00 \|  \| \| Onion seed  Kamal kakdi  Ginger  Curry leaves  Ansi flower \| 0.00  0.01  0.07  0.03  0.00 \|  \| \| Potato \| 1.00 \| Potato \| \| Colacasia \| 0.63 \| Starchy roots \| \| Yam \| 0.37 \|  \| \| Tomatoes \| 1.00 \| Veg - tomato \| \| Tomato puree \| 0.00 \|  \| \| Bitter gourd \| 0.04 \| Veg - gourd \| \| Bottle gourd \| 0.57 \|  \| \| Dhemsa/tinda \| 0.10 \|  \| \| Lauki \| 0.03 \|  \| \| Kundru \| 0.10 \|  \| \| Parwal \| 0.13 \|  \| \| Ridge gourd \| 0.03 \|  \| \| Carrot \| 1.00 \| Veg - carrot \| \| Cauliflower \| 0.12 \| Veg - other \| \| Ladies finger \| 0.09 \|  \| \| Brinjal \| 0.12 \|  \| \| Cucumber \| 0.07 \|  \| \| Red pumpkin \| 0.06 \|  \| \| Capsicum \| 0.04 \|  \| \| Drum stick \| 0.04 \|  \| \| Mushroom \| 0.00 \|  \| \| Green beans  Beetroot  Radish  Turnip  Chow chow marrow  Green chilli \| 0.09  0.05  0.17  0.00  0.01  0.14 \|  \| \| Coconut oil \| 0.00 \| Veg oils \| \| Mustard oil \| 0.25 \|  \| \| Soya oil \| 0.21 \|  \| \| Groudnut oil \| 0.16 \|  \| \| Dalda \| 0.03 \|  \| \| Sunflower oil \| 0.34 \|  \| \| Palm oil  Rice bran oil  Oilive oil \| 0.01  0.00  0.00 \|  \| |
| --- | --- | --- | --- | --- | --- | --- | --- | --- | --- | --- | --- | --- | --- | --- | --- | --- | --- | --- | --- | --- | --- | --- | --- | --- | --- | --- | --- | --- | --- | --- | --- | --- | --- | --- | --- | --- | --- | --- | --- | --- | --- | --- | --- | --- | --- | --- | --- | --- | --- | --- | --- | --- | --- | --- | --- | --- | --- | --- | --- | --- | --- | --- | --- | --- | --- | --- | --- | --- | --- | --- | --- | --- | --- | --- | --- | --- | --- | --- | --- | --- | --- | --- | --- | --- | --- | --- | --- | --- | --- | --- | --- | --- | --- | --- | --- | --- | --- | --- | --- | --- | --- | --- | --- | --- | --- | --- | --- | --- | --- | --- | --- | --- | --- | --- | --- | --- | --- | --- | --- | --- | --- | --- | --- | --- | --- | --- | --- | --- | --- | --- | --- | --- | --- | --- | --- | --- | --- | --- | --- | --- | --- | --- | --- | --- | --- | --- | --- | --- | --- | --- | --- | --- | --- | --- | --- | --- | --- | --- | --- | --- | --- | --- | --- | --- | --- | --- | --- | --- | --- | --- | --- | --- | --- | --- | --- | --- | --- | --- | --- | --- | --- | --- | --- | --- | --- | --- | --- | --- | --- | --- | --- | --- | --- | --- | --- | --- | --- | --- | --- | --- | --- | --- | --- | --- | --- | --- | --- | --- | --- | --- | --- | --- | --- | --- | --- | --- | --- | --- | --- | --- | --- | --- | --- | --- | --- | --- | --- | --- | --- | --- | --- | --- | --- | --- | --- | --- | --- | --- | --- | --- | --- | --- | --- | --- | --- | --- | --- | --- | --- | --- | --- | --- | --- | --- | --- | --- | --- | --- | --- | --- | --- | --- | --- | --- | --- | --- | --- | --- | --- | --- | --- | --- | --- | --- | --- | --- | --- | --- | --- | --- | --- | --- | --- | --- | --- | --- | --- | --- | --- | --- | --- | --- | --- | --- | --- | --- | --- | --- | --- | --- | --- | --- | --- | --- | --- | --- | --- | --- | --- | --- | --- | --- | --- | --- | --- | --- | --- | --- | --- | --- | --- | --- | --- | --- | --- | --- | --- | --- | --- | --- | --- | --- | --- | --- | --- | --- | --- | --- | --- | --- | --- | --- | --- | --- | --- | --- | --- | --- | --- | --- | --- | --- | --- | --- | --- | --- | --- | --- | --- | --- | --- | --- | --- | --- | --- | --- | --- | --- | --- | --- | --- | --- | --- | --- | --- | --- | --- | --- | --- | --- | --- | --- | --- | --- | --- | --- | --- | --- | --- | --- | --- | --- | --- | --- | --- | --- | --- | --- | --- | --- | --- | --- | --- | --- | --- | --- | --- | --- | --- | --- | --- | --- | --- | --- | --- | --- | --- | --- | --- | --- | --- | --- | --- | --- | --- | --- | --- | --- | --- | --- | --- | --- | --- | --- | --- | --- | --- | --- | --- | --- | --- | --- | --- | --- | --- | --- | --- | --- | --- | --- | --- | --- | --- | --- | --- | --- | --- | --- | --- | --- | --- | --- | --- | --- | --- | --- | --- | --- | --- | --- | --- | --- | --- | --- | --- | --- | --- | --- | --- | --- | --- | --- | --- | --- | --- | --- | --- | --- | --- | --- | --- | --- | --- | --- | --- | --- | --- | --- | --- | --- | --- | --- | --- | --- | --- | --- | --- | --- | --- | --- | --- | --- | --- | --- | --- | --- | --- | --- | --- | --- | --- | --- | --- | --- | --- | --- | --- | --- | --- | --- | --- | --- | --- | --- | --- | --- | --- | --- | --- | --- | --- | --- | --- | --- | --- | --- | --- | --- | --- | --- | --- | --- | --- | --- | --- |

# Table A2. Socioeconomic characteristics of respondents in the Indian Migration Study (2005–7) by dietary pattern.

|  | Rice & low diversity | Rice & fruit | Wheat & pulses | Wheat, rice & oils | Rice & meat | Total |
| --- | --- | --- | --- | --- | --- | --- |
|  |  | | | | | |
|  | n (%) | | | | | |
|  |  |  |  |  |  |  |
|  | 1339 (19.8) | 1505 (22.2) | 1953 (28.8) | 1462 (21.6) | 516 (7.6) | 6775 |
|  |  |  |  |  |  |  |
| *Sex* |  |  |  |  |  |  |
| Males | 784 (58.5) | 724 (48.1) | 1172 (60.0) | 888 (60.7) | 312 (60.5) | 3880 (57.3) |
| Females | 555 (41.5) | 781 (52.9) | 781 (40.0) | 574 (39.3) | 204 (39.5) | 2895 (42.7) |
|  |  |  |  |  |  |  |
| *Age* |  |  |  |  |  |  |
| Mean (SD) | 44 (9.5) | 44 (8.6) | 44 (8.8) | 30 (7.3) | 44 (10.2) | 41 (10.3) |
|  |  |  |  |  |  |  |
| *Region* |  |  |  |  |  |  |
| North | 6 (0.5) | 6 (0.4) | 1837 (94.1) | 178 (12.2) | 9 (1.7) | 2036 (30.0) |
| East | 11 (0.8) | 5 (0.3) | 54 (2.8) | 52 (3.6) | 3 (0.6) | 125 (1.85) |
| South | 1302 (97.2) | 1477 (98.2) | 11 (0.5) | 31 (2.1) | 471 (91.3) | 3292 (48.6) |
| West | 20 (1.5) | 17 (1.1) | 51 (2.6) | 1201 (82.1) | 33 (6.4) | 1322 (19.5) |
|  |  |  |  |  |  |  |
| *Location* |  |  |  |  |  |  |
| Urban | 573 (42.8) | 1163 (77.3) | 1346 (68.9) | 910 (62.2) | 277 (53.7) | 4269 (63.0) |
| Rural | 766 (57.2) | 342 (22.7) | 607 (31.1) | 552 (37.8) | 239 (46.3) | 2506 (37.0) |
|  |  |  |  |  |  |  |
| *Marital status* |  |  |  |  |  |  |
| Married | 1206 (90.1) | 1387(92.2) | 1802 (92.3) | 1092 (74.7) | 457 (88.6) | 5944 (87.7) |
| Unmarried | 133 (9.9) | 118 (7.8) | 151 (7.7) | 370 (25.3) | 59 (11.4) | 831 (12.3) |
|  |  |  |  |  |  |  |
| *Education* |  |  |  |  |  |  |
| None | 325 (24.3) | 92 (6.1) | 173 (8.9) | 53 (3.6) | 123 (23.8) | 766 (11.3) |
| Primary | 299 (22.3) | 229(15.2) | 198 (10.1) | 84 (5.8) | 102 (19.8) | 912 (13.5) |
| Secondary | 557 (41.6) | 729 (48.5) | 749 (38.3) | 983 (67.2) | 230 (44.6) | 3248 (47.9) |
| Tertiary | 158 (11.8) | 455 (30.2) | 833 (42.7) | 342 (23.4) | 61 (11.8) | 1849 (27.3) |
|  |  |  |  |  |  |  |
| *Occupation* |  |  |  |  |  |  |
| None | 447(33.4) | 614 (40.8) | 732 (37.5) | 613 (41.9) | 165 (32.0) | 2571 (38.0) |
| Unskilled manual | 306 (22.8) | 114 (7.6) | 353 (18.1) | 274 (18.7) | 108 (20.9) | 1155 (17.1) |
| Skilled manual | 296 (22.1) | 330 (21.9) | 201 (10.3) | 482 (33.0) | 127 (24.6) | 1436 (21.2) |
| Non-manual | 229(17.1) | 203 (13.5) | 557 (28.5) | 46 (3.2) | 93 (18.0) | 1128 (16.7) |
| Professional | 61 (4.6) | 244 (16.2) | 110 (5.6) | 47 (3.2) | 23 (4.5) | 485 (7.2) |
|  |  |  |  |  |  |  |
| *Religion* |  |  |  |  |  |  |
| Hindu | 1216 (90.8) | 1383 (91.9) | 1799 (92.1) | 1342 (91.8) | 434 (84.1) | 6174 (91.1) |
| Other | 123 (9.2) | 122 (8.1) | 154 (7.9) | 120 (8.2) | 82 (15.9) | 601 (8.9) |
|  |  |  |  |  |  |  |
| *Own agricultural land* |  |  |  |  |  |  |
| Yes | 692 (51.7) | 533 (35.4) | 691 (35.4) | 545 (37.3) | 233 (45.2) | 2692 (39.8) |
| No | 647 (48.3) | 972 (64.6) | 1262 (64.6) | 917 (62.7) | 283 (54.8) | 4081 (60.2) |

**Table A3. Domestic supply and imports of major food groups in India, from FAOSTAT (excluding alcoholic beverages)**

| **Food group** | **Domestic supply (100 metric tons)** | |  |
| --- | --- | --- | --- |
|  | *Total* | *Imported* | *Percent imported* |
| Cereals | 216517 | 113 | 0.1 |
| Starchy roots | 53555 | 103 | 0.2 |
| Sugar crops | 341200 | 0 | 0.0 |
| Sugar and sweeteners | 32578 | 1347 | 4.1 |
| Pulses | 21685 | 3801 | 17.5 |
| Treenuts | 1975 | 1064 | 53.9 |
| Oilcrops | 49765 | 146 | 0.29 |
| Vegetable oils | 18820 | 11159 | 59.3 |
| Vegetables | 118525 | 56 | 0.1 |
| Fruits | 82268 | 779 | 1.0 |
| Stimulants | 1025 | 127 | 12.4 |
| Spices | 3474 | 115 | 3.3 |
| Meat | 4627 | 1 | 0.0 |
| Offals | 475 | 0 | 0.0 |
| Animal fats | 4026 | 2 | 0.1 |
| Eggs | 3763 | 0 | 0.0 |
| Milk | 133443 | 18 | 0.0 |
| Seafood | 6992 | 50 | 0.7 |
| Aquatic products | 4 | 0 | 0.0 |

Data taken from FAOSTAT (<http://faostat3.fao.org/home/E>)

**Table A4. Estimation of GHGs for 36 food groups from IMS data, with data sources**

| *Food Group* | *CO_2_ equivalent emissions in kg per kg food for each stage of production process* | | | | | | | | |
| --- | --- | --- | --- | --- | --- | --- | --- | --- | --- |
|  | **Primary production** | **Waste (production)** | **Edible production*** | **Storage** | **Transport** | **Processing & packaging** | **Cooking** | **Waste (household)** | **TOTAL**** |
| *Cereals – wheat* | 0.30^1^ | 0.00^4^ | 0.27^4^ | 0.04^5^ | 0.01^2^ | 0.10^6^ | 0.04^4^ | 0.09^9^ | 0.54 |
| *Cereals – rice* | 0.73^1^ | 0.00^4^ | 1.11^4^ | 0.04^5^ | 0.01^2^ | 0.16^6^ | 0.04^2^ | 0.27^9^ | 1.61 |
| *Cereals - other* | 0.43^1^ | 0.00^4^ | 0.46^4^ | 0.04^5^ | 0.01^2^ | 0.10^6^ | 0.04^4^ | 0.13^9^ | 0.77 |
| *Dairy – lo-fat* | 1.93^1^ | 0.02^4^ | 1.95^4^ | 0.00^5^ | 0.02^2^ | 0.12^6^ | 0.00^2^ | 0.44^9^ | 2.52 |
| *Dairy – hi-fat* | 1.93^1^ | 0.02^4^ | 4.64^4^ | 0.01^5^ | 0.02^2^ | 1.00^6^ | 0.00^2^ | 1.19^9^ | 6.85 |
| *Dairy – butter/ghee* | 1.71^1^ | 0.02^4^ | 9.67^4^ | 0.01^5^ | 0.02^2^ | 0.66^6^ | 0.00^2^ | 2.18^9^ | 12.53 |
| *Fish, seafood* | 0.58^2^ | 0.00^4^ | 0.67^4^ | 0.00^5^ | 0.02^2^ | 0.10^6^ | 0.09^2^ | 0.30^9^ | 1.17 |
| *Fruit – mango* | 0.03^1^ | 0.00^4^ | 0.03^4^ | 0.02^5^ | 0.03^2^ | 0.00^6^ | 0.00^2^ | 0.04^9^ | 0.11 |
| *Fruit – orange* | 0.12^1^ | 0.01^4^ | 0.13^4^ | 0.02^5^ | 0.03^2^ | 0.00^6^ | 0.00^2^ | 0.09^9^ | 0.26 |
| *Fruit – guava* | 0.03^1^ | 0.01^4^ | 0.03^4^ | 0.02^5^ | 0.03^2^ | 0.00^6^ | 0.00^2^ | 0.04^9^ | 0.12 |
| *Fruit – banana* | 0.07^2^ | 0.01^4^ | 0.09^4^ | 0.02^5^ | 0.03^2^ | 0.00^6^ | 0.00^2^ | 0.07^9^ | 0.20 |
| *Fruit – papaya* | 0.03^1^ | 0.01^4^ | 0.03^4^ | 0.02^5^ | 0.03^2^ | 0.00^6^ | 0.00^2^ | 0.04^9^ | 0.12 |
| *Fruit – grapes* | 0.31^2^ | 0.03^4^ | 0.35^4^ | 0.02^5^ | 0.03^2^ | 0.00^6^ | 0.00^2^ | 0.20^9^ | 0.59 |
| *Fruit – melon* | 0.31^2^ | 0.05^4^ | 0.36^4^ | 0.02^5^ | 0.03^2^ | 0.00^6^ | 0.00^2^ | 0.21^9^ | 0.61 |
| *Fruit – other* | 0.03^1^ | 0.01^4^ | 0.08^4^ | 0.02^5^ | 0.03^2^ | 0.00^6^ | 0.00^2^ | 0.04^9^ | 0.12 |
| *Meat – poultry* | 0.78^1^ | 0.00^4^ | 0.90^4^ | 0.00^5^ | 0.02^2^ | 0.00^6^ | 0.27^2^ | 0.24^9^ | 1.43 |
| *Egg* | 0.78^1^ | 0.00^4^ | 0.81^4^ | 0.00^5^ | 0.02^2^ | 0.00^6^ | 0.10^2^ | 0.19^9^ | 1.12 |
| *Meat – mutton* | 39.9^1^ | 0.00^4^ | 71.03^4^ | 0.00^5^ | 0.02^2^ | 0.00^6^ | 0.26^2^ | 14.26^9^ | 85.57 |
| *Meat – other* | 0.78^1^ | 0.00^4^ | 0.90^4^ | 0.00^5^ | 0.02^2^ | 0.00^6^ | 0.27^2^ | 0.24^9^ | 1.43 |
| *Legumes* | 0.62^1^ | 0.02^4^ | 1.03^4^ | 0.04^5^ | 0.08^2^ | 0.00^6^ | 0.24^2^ | 0.37^9^ | 1.76 |
| *Nuts and seeds* | 0.88^3^ | 0.02^4^ | 0.90^4^ | 0.04^5^ | 0.08^2^ | 0.00^6^ | 0.00^2^ | 0.27^9^ | 1.29 |
| *Other – sugar* | 0.03^1^ | 0.00^4^ | 0.27^4^ | 0.04^5^ | 0.01^2^ | 0.10^7^ | 0.00^2^ | 0.08^9^ | 0.50 |
| *Other* | 1.43^1^ | 0.00^4^ | 1.43^4^ | 0.04^5^ | 0.02^2^ | 1.00^8^ | 0.14^8^ | 0.52^9^ | 3.14 |
| *Pulses – red gram* | 0.76^1^ | 0.02^4^ | 0.82^4^ | 0.04^5^ | 0.01^2^ | 0.00^6^ | 0.24^2^ | 0.30^9^ | 1.40 |
| *Salt* | 1.00^3^ | 0.00^4^ | 1.00^4^ | 0.04^5^ | 0.01^2^ | 0.57^8^ | 0.00^2^ | 0.32^9^ | 1.94 |
| *Pulses – other* | 0.57^1^ | 0.06^4^ | 0.63^4^ | 0.04^5^ | 0.01^2^ | 0.00^6^ | 0.24^2^ | 0.25^9^ | 1.16 |
| *Veg – onion and garlic* | 0.39^1^ | 0.02^4^ | 0.41^4^ | 0.02^5^ | 0.01^2^ | 0.00^6^ | 0.05^2^ | 0.25^9^ | 0.74 |
| *Leafy veg* | 0.02^1^ | 0.00^4^ | 0.02^4^ | 0.02^5^ | 0.01^2^ | 0.00^6^ | 0.05^2^ | 0.05^9^ | 0.16 |
| *Spices – other* | 0.75^1^ | 0.00^4^ | 0.75^4^ | 0.02^5^ | 0.01^2^ | 0.00^6^ | 0.05^2^ | 0.42^9^ | 1.25 |
| *Potato* | 0.07^1^ | 0.01^4^ | 0.09^4^ | 0.00^5^ | 0.01^2^ | 0.00^6^ | 0.25^2^ | 0.15^9^ | 0.50 |
| *Starchy roots* | 0.88^3^ | 0.04^4^ | 0.92^4^ | 0.00^5^ | 0.01^2^ | 0.00^6^ | 0.25^2^ | 0.49^9^ | 1.68 |
| *Veg – tomato* | 0.01^1^ | 0.00^4^ | 0.01^4^ | 0.02^5^ | 0.01^2^ | 0.00^6^ | 0.05^2^ | 0.05^9^ | 0.14 |
| *Veg – gourd* | 0.05^1^ | 0.01^4^ | 0.06^4^ | 0.02^5^ | 0.01^2^ | 0.00^6^ | 0.05^2^ | 0.07^9^ | 0.21 |
| *Veg – carrot* | 0.35^3^ | 0.04^4^ | 0.39^4^ | 0.02^5^ | 0.01^2^ | 0.00^6^ | 0.05^2^ | 0.24^9^ | 0.70 |
| *Veg – other* | 0.05^1^ | 0.00^4^ | 0.05^4^ | 0.02^5^ | 0.01^2^ | 0.00^6^ | 0.05^2^ | 0.07^9^ | 0.20 |
| *Veg oils* | 0.62^1^ | 0.00^4^ | 0.62^4^ | 0.02^5^ | 0.02^2^ | 0.16^6^ | 0.00^2^ | 0.22^9^ | 1.04 |

* Edible production emissions calculated using the formula: edible production = (production+waste)*(value fraction/product fraction). The product fraction describes the proportion of the product that can be eaten, while the value fraction describes the proportional value of the food group compared to the value of all products that can be made from that crop.

**Total emissions calculated by adding Edible Production, Storage, Transport, Processing & Packaging, Cooking and Household Waste.

**^1^** Vetter *et al* 2016 (extrapolated from most similar food group where necessary)

^2^ Pathak *et al* 2010 (extrapolated from most similar food group where necessary)

^3^ Audsley *et al* 2009 (extrapolated from most similar food group where necessary)

^4^FAO Technical Conversion Factors 1972

^5^ Roos 2015 (extrapolated from most similar food group where necessary)

^6^ FoodCarbonScope CleanMetrics software <http://www.cleanmetrics.net/foodcarbonscope/DefineModelDistrib.aspx> (accessed June 2018)

^7^ Rein 2010

^8^ Foster 2006 (extrapolated from most similar food group where necessary)

^9^ Food waste data extrapolated from FAO 2011 using factors for South and South East Asia (20% for cereals, 41% for starchy roots, 27% for oilcrops and pulses, 51% for fruits, 20% for meat, 34% for fish and seafood, 21% for milk and dairy, 51% for vegetables)

**Table A5. Comparison between national-level and state-level data for environmental impacts**

| **Dietary pattern** | **GHG emissions** | | **Water footprint** | | | |
| --- | --- | --- | --- | --- | --- | --- |
|  |  |  | **Green** | **Blue** | **Green** | **Blue** |
|  | **kg CO_2_e *capita*^-1^ d^-1^** | **g CO_2_e kcal^-1^** | **L d^-1^** | | **L kcal^-1^** | |
| **National-level data** |  |  |  |  |  |  |
| Rice & low diversity | 4.01 (1.46) | 1.71 (0.35) | 2209 (797) | 566 (208) | 0.93 (0.13) | 0.24 (0.04) |
| Rice & fruit | 3.88 (1.24) | 1.42 (0.28) | 2683 (924) | 640 (191) | 0.96 (0.12) | 0.23 (0.03) |
| Wheat & pulses | 2.99 (1.12) | 0.99 (0.25) | 2492 (820) | 836 (252) | 0.82 (0.11) | 0.28 (0.03) |
| Wheat, rice & oils | 3.19 (1.17) | 0.95 (0.24) | 2636 (864) | 883 (254) | 0.79 (0.13) | 0.26 (0.03) |
| Rice & meat | 4.64 (1.73) | 1.73 (0.47) | 2776 (1032) | 677 (248) | 1.01 (0.15) | 0.25 (0.03) |
| All | 3.56 (1.39) | 1.28 (0.44) | 2531 (885) | 737 (263) | 0.88 (0.15) | 0.25 (0.04) |
|  |  |  |  |  |  |  |
| **State-level data** |  |  |  |  |  |  |
| Rice & low diversity | 3.31 (1.33) | 1.40 (0.34) | 2252 (847) | 611 (242) | 0.95 (0.14) | 0.26 (0.05) |
| Rice & fruit | 3.68 (1.26) | 1.34 (0.28) | 2914 (1057) | 741 (241) | 1.04 (0.15) | 0.27 (0.04) |
| Wheat & pulses | 2.74 (1.08) | 0.91 (0.24) | 2349 (823) | 814 (283) | 0.78 (0.12) | 0.27 (0.04) |
| Wheat, rice & oils | 3.79 (1.40) | 1.13 (0.29) | 2780 (902) | 1242 (418) | 0.83 (0.14) | 0.37 (0.08) |
| Rice & meat | 4.01 (1.63) | 1.48 (0.43) | 2829 (1109) | 734 (306) | 1.03 (0.17) | 0.27 (0.05) |
| All | 3.18 (1.29) | 1.19 (0.36) | 2585 (964) | 844 (375) | 0.90 (0.18) | 0.29 (0.07) |

***A1. Sensitivity analysis***

Sensitivity to variation in production factors was investigated by matching individuals’ consumption of food groups to state-specific data on GHG emissions and WFs of food items, based on the residency location of IMS participants (Table 1). Use of state-level data resulted in lower emissions associated with all dietary patterns except W*heat, rice & oils*. Over 77% of individuals in the *Rice & low diversity* and *Rice & meat* dietary patterns resided in Andhra Pradesh where emissions associated with rice production were 2.7 kg CO_2_e kg^-1^ of rice compared to 5.7 kg CO_2_e kg^-1^ at a national level, leading to ~15% lower emissions associated with diets. Similarly, the majority of individuals in the *Wheat & pulses* pattern resided in Uttar Pradesh where emissions associated with rice production were 2.4 kg CO_2_e kg^-1^ of rice – since rice consumption was relatively low in this pattern, use of state-level data only resulted in 8% lower emissions associated with the diet. Of individuals in the *Rice & fruit* pattern, 65% resided in Karnataka where emissions associated with rice production were 5.8 kg CO_2_e kg^-1^ of rice (i.e. very similar to national level); however, 31% resided in Andhra Pradesh and emissions associated with diets were 5% lower using state-level data. Of individuals in the *Wheat, rice & oils* pattern, 82% resided in Maharashtra where emissions associated with rice production were 9.2 kg CO_2_e kg^-1^ of rice leading to 19% greater emissions associated with diets when using state-level data. Differences between state and national-level data in the emissions associated with wheat production had little effect on the overall emissions associated with diets because wheat contributed little to emissions.

The green and blue WFs across dietary patterns increased by 2% and 15%, respectively, when food consumption data were matched to state-specific WF data rather than national-level data. Similar changes were found in the WFs kcal^-1^. The relative differences in green WF kcal^-1^ between the dietary patterns remained broadly the same, with greater WFs for rice-based than wheat-based patterns. The relative differences in blue WF kcal^-1^ between the dietary patterns became less distinct except for the *Wheat, rice & oils* pattern which increased from 0.26±0.03 L kcal^-1^ to 0.37 L kcal^-1^. Over 80% of participants in the *Wheat, rice & oils* pattern resided in Maharashtra. The blue WF for wheat in this state was 2.69 L g^-1^ compared to 1.36 L g^-1^ nationally; thus, the blue WF of wheat consumption ~doubled for the majority of individuals in the *Wheat, rice & oils* pattern when state-specific rather than national data were used.
